# Effect of lymphadenectomy on the prognosis for N0 gallbladder carcinoma patients: A study based on SEER database

**DOI:** 10.1002/cam4.4250

**Published:** 2021-09-14

**Authors:** Bin Wu, Yiyu Shen, Xujian Chen, Xiaoguang Wang, Zhengxiang Zhong

**Affiliations:** ^1^ Department of Hepatobiliary Surgery The Second Affiliated Hospital of Jiaxing University Jiaxing P.R. China

**Keywords:** lymphadenectomy, N0 gallbladder carcinoma, prognosis

## Abstract

**Background:**

It remains unclear whether lymph node dissection is necessary for patients with N0 gallbladder carcinoma (GBC). The objective of this study was to evaluate the effect of lymphadenectomy on the prognosis for N0 GBC patients. The secondary objective was to establish a prognostic model of survival for N0 GBC patients being founded on the large samples.

**Methods:**

Patient data were obtained from the database named SEER (Surveillance, Epidemiology, and End Results database) between 2010 and 2014. Analyses of Kaplan–Meier survival and multivariate Cox regression were performed in subgroups based on regional lymph nodes removal (LNR) to calculate the excess risk of cause‐specific death. A prognosis nomogram was constructed build on the results of a multivariate analysis to predict the specific survival time (CSS) rates of N0 GBC patients.

**Result:**

A total of 1406 N0 GBC patients were included in this research. The majority of N0 GBC patients undergoing cancer‐directed surgery did not undergo LNR (64.5%). The results showed that LNR can improve the survival of N0 GBC patients, including those at the T1a and T1b stages, and a wider range of lymph node dissection (LNR2) compared to LNR1 was more conducive to the prognosis. Furthermore, multivariate regression analysis showed that LNR was an independent favorable prognostic factor of N0 GBC. Finally, a nomogram was constructed to accurately predict the prognosis of N0 gallbladder cancer patients.

**Conclusion:**

This study demonstrated a significant survival benefit for extended lymph nodes removed in N0 GBC patients. These results recommend that an extended lymph node dissection strategy is needed for N0 GBC patients.

## Graphical abstract

Our study demonstrated a significant survival benefit for extended lymph nodes removed in N0 gallbladder carcinoma (GBC) patients. These results recommend that an extended lymph node dissection strategy is needed for N0 GBC patients.

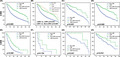

## 1 INTRODUCTION

Gallbladder carcinoma (GBC) is a more malignant neoplasm with a very poor prognosis, and the 5‐year overall survival rate was <5%.^1^ The presentation characteristics of GBC are nonspecific, and the clinical symptoms are indistinguishable from other disorders. The incidence has increased year by year with routine physical examination and laparoscopic cholecystectomy being performed widely.^2^, ^3^

Lymph node metastasis, as the main pathway of GBC tumor metastasis, is an important prognostic factor. Radical surgery is widely recognized as the main treatment of GBC.^4^ As early as 1954, Glenn and Jays first proposed radical surgery, which included cholecystectomy and lymph node dissection, for extrahepatic biliary malignancies.^5^ Subsequently, Pack et al. advocated a more aggressive treatment for GBC, with a combination of complete right hepatectomy and lymph node dissection.^5^, ^6^ Currently, the NCCN (National Comprehensive Cancer Network) recommends radical cholecystectomy and regional lymph node dissection, including retroduodenal lymph nodes, hepatoduodenal ligament lymph nodes, porta hepatis lymph nodes, and en bloc hepatic resection.^7^ For GBC patients with lymph node metastasis, most guidelines reach a consensus that lymph node dissection should be necessary.^8^ However, the range of lymph node dissection, as well as lymph node dissection for patients without lymph node metastasis, is still controversial,^9^ especially for T1aN0 and T1bN0 GBC patients.

The NCCN guidelines clearly indicate that lymphadenectomy is recommended for gallbladder cancer patients with positive lymph nodes in the N1 region (porta hepatis lymph node). Lymph node dissection is not recommended for patients with positive N2 lymph nodes (superior mesenteric artery, aortocaval, and celiac axis lymph nodes).^10^, ^11^ However, there is no clear guidance on the need for prophylactic lymph node dissection in patients with N0 stage (negative for regional lymph node pathology). In the current clinical work, sentinel lymph node pathology plays a decisive role in our surgical selection, and we usually do not take further lymph node dissection for these patients.^12^ However, studies support the benefit of prophylactic extensive lymph node dissection in patients with gallbladder cancer.^13^, ^14^ This can not only conduct N staging more accurately to guide treatment, but also prevent the occurrence of insidious diseases. Because clinically diagnosed “cN0” patients or “pN0” patients with negative sentinel lymph node pathology may be mixed with N+ patients.^15^, ^16^ Therefore, in the Japanese study, extended lymphadenectomy was recommended, and the number of lymph nodes to be removed was 12–22.^13^

In this study, a large sample of patients with GBC was obtained from the Surveillance, Epidemiology, and End Results (SEER) database, which named the National Cancer Institute's Surveillance, Epidemiology, and End Results database, to investigate the effect of regional lymphadenectomy, also known as regional lymph nodes removal or LNR, on the prognosis for N0 GBC patients and explore the necessity for prophylactic lymph node dissection in such patients. In addition, we construct a prognostic model of survival of N0 GBC patients based on the large samples.

## 2 PATIENTS AND METHODS

### 2.1 Study population and data sources

The SEER stat software^17^ (version 8.3.2) was used to recognize N0 patients who received a pathological diagnosis of GBC between 2010 and 2014. Histological type was limited to adenocarcinoma (8140/3) according to ICD‐O‐3.^18^ TNM classification was performed according to the criteria of the AJCC Cancer Staging Manual (The seventh edition, 2010). Patients were excluded if they had incomplete information about TNM staging, grade, liver metastasis, regional LNR, survival months, and survival status. Patients diagnosed within 1 month prior to death were excluded from the survival analysis because the survival time in the SEER database is measured in months rather than days, so these cases would be considered to have a zero survival.^19^ Regional LNR is divided into three categories: 1–3 regional lymph nodes removed (LNR1), 4 or more regional lymph nodes removed (LNR2), and no regional lymph nodes removed (Non‐LNR).

### 2.2 Statistical analysis

#### 2.2.1 Survival analyses

The analysis used cause‐specific death as the follow‐up endpoint, and the cancer‐specific survival time (CSS) as the survival outcome. Survival curves were described by method of Kaplan–Meier and the survival differences between the curves were analyzed by analysis of log‐rank test. Univariate and multivariate Cox regression analyses were performed for the risk factors analysis for survival outcomes. The above calculations and analyses were performed by SPSS software (version 22.0), which was published by IBM Corp.

#### 2.2.2 Variables

The data of patients’ clinicpathological characteristics, such as race, sex, age at diagnosis, T stage, M stage, grade, liver metastasis, surgery, and regional LNR, were collected.

#### 2.2.3 Nomogram

Based on the results of multivariate analysis, a nomogram was constructed using R software version 3.4.1 with the rms package.^19^ The consistency index (*C* index) was used to evaluate the performance of the nomogram. This method is evaluated by comparing the prediction of observed Kaplan–Meier estimates of survival probability versus nomogram‐predicted. These analyses were performed by bootstraps of 1000 resamples. The larger *C* index, the higher accuracy of prognosis prediction. Finally, the calibration curve and *C*‐index were obtained by the regression analysis. *p* value < 0.05 was considered statistically significant.

## 3 RESULTS

### 3.1 Patient characteristics

A total of 1560 N0 GBC patients were identified in the SEER database. One hundred and fifty‐four patients with incomplete data were excluded and a total of 1406 patients with GBC were enrolled in the study. Table 1 shows the clinical characteristics of the included GBC patients. Of all of the N0 GBC patients, 907 had no regional lymph node removed (Non‐LNR), and 499 had regional LNR, including 322 cases of 1–3 regional lymph nodes (LNR1) and 177 cases of 4 or more regional lymph nodes removed (LNR2).

**TABLE 1:** Clinicopathological characteristics of N0 gallbladder carcinoma patients

Variable	Non‐LNR^a^ (%)	LNR1^a^ (%)	LNR2^a^ (%)
No. of patients (*n*)	907	322	177
Median age (years)	74	65	69
Sex, *n* (%)			
Male	275 (30.3)	98 (30.4)	65 (36.7)
Female	632 (69.7)	224 (69.6)	112 (63.3)
Race, *n* (%)			
White	701 (77.3)	253 (78.6)	130 (73.4)
Black	110 (12.1)	43 (13.4)	27 (15.3)
Other	96 (10.6)	26 (8.1)	20 (11.3)
AJCC stage			
Ⅰ	156 (17.2)	60 (18.6)	20 (11.3)
Ⅱ	360 (39.7)	167 (51.9)	105 (59.3)
Ⅲ	200 (22.1)	69 (21.4)	39 (22.0)
Ⅳ	191 (21.1)	26 (8.1)	13 (7.3)
T			
T1	171 (18.9)	61 (18.9)	21 (11.9)
T2	424 (46.7)	176 (54.7)	108 (61.0)
T3	299 (33.0)	81 (25.2)	46 (26.0)
T4	13 (1.4)	4 (1.2)	2 (1.1)
M			
M0	726 (80.0)	300 (93.2)	164 (92.7)
M1	181 (20.0)	22 (6.8)	13 (7.3)
Grade, *n* (%)			
Well differentiated I	166 (18.3)	66 (20.5)	40 (22.6)
Moderately differentiated II	412 (45.4)	169 (52.5)	103 (58.2)
Poorly or undifferentiated III/IV	329 (36.3)	87 (27.0)	34 (19.2)
Liver metastasis			
No	797 (87.9)	306 (95.0)	167 (94.4)
Yes	110 (12.1)	16 (5.0)	10 (5.6)

^a^ Non‐LNR: no regional lymph nodes removed; LNR1: 1–3 regional lymph nodes removed; LNR2: 4 or more regional lymph nodes removed.

**FIGURE 1:**
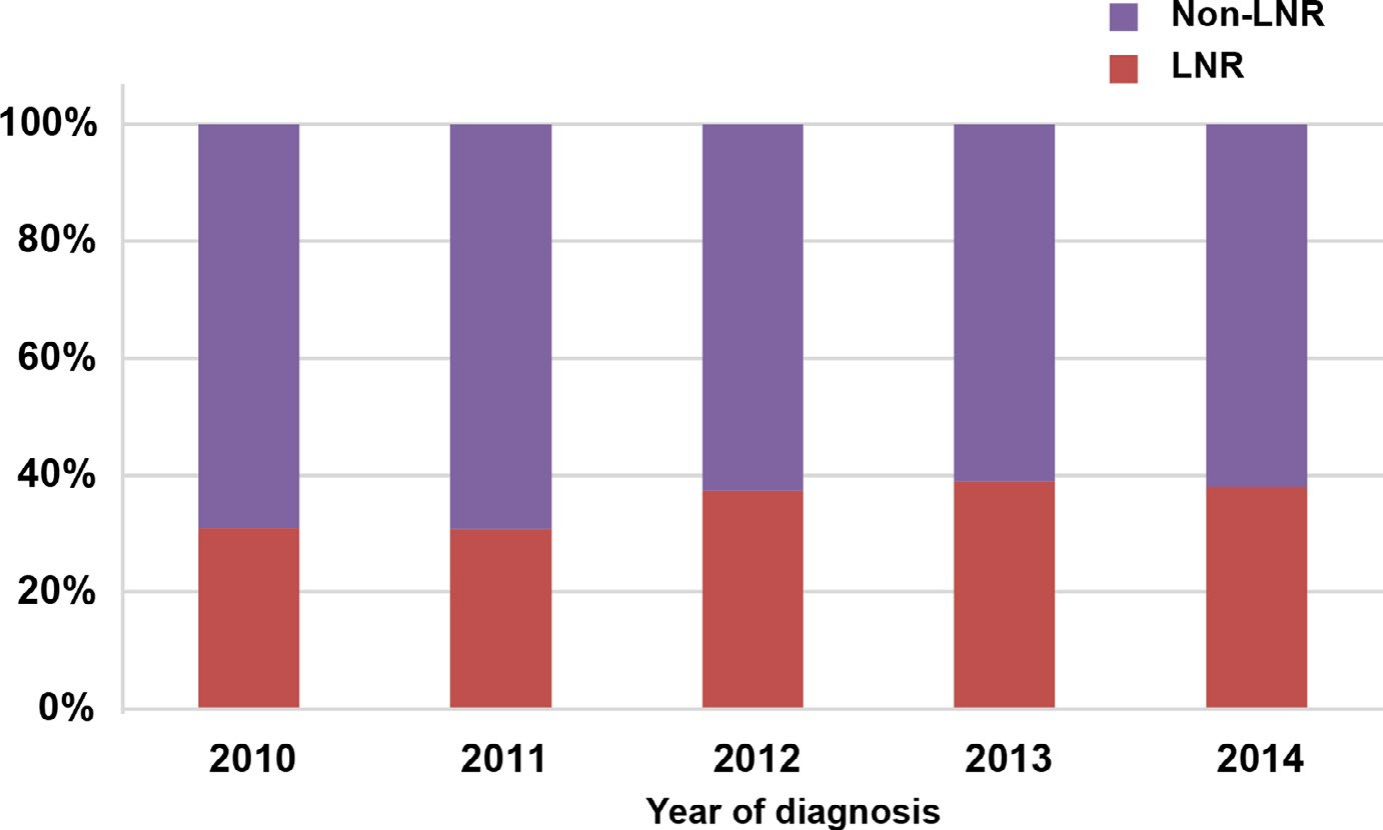
Trend in number of lymph nodes removed by year of diagnosis. LNR, lymph nodes removal

The majority did not undergo LNR (64.5%) in N0 GBC patients with tumor‐related surgery. Although, the percentage of GBC patients undergoing LNR in 2014 increased by approximately 10% over 2010, and the percentage remained only 38.1% (Figure 1).

### 3.2 Effect of LNR on CSS in N0 GBC patients

**FIGURE 2:**
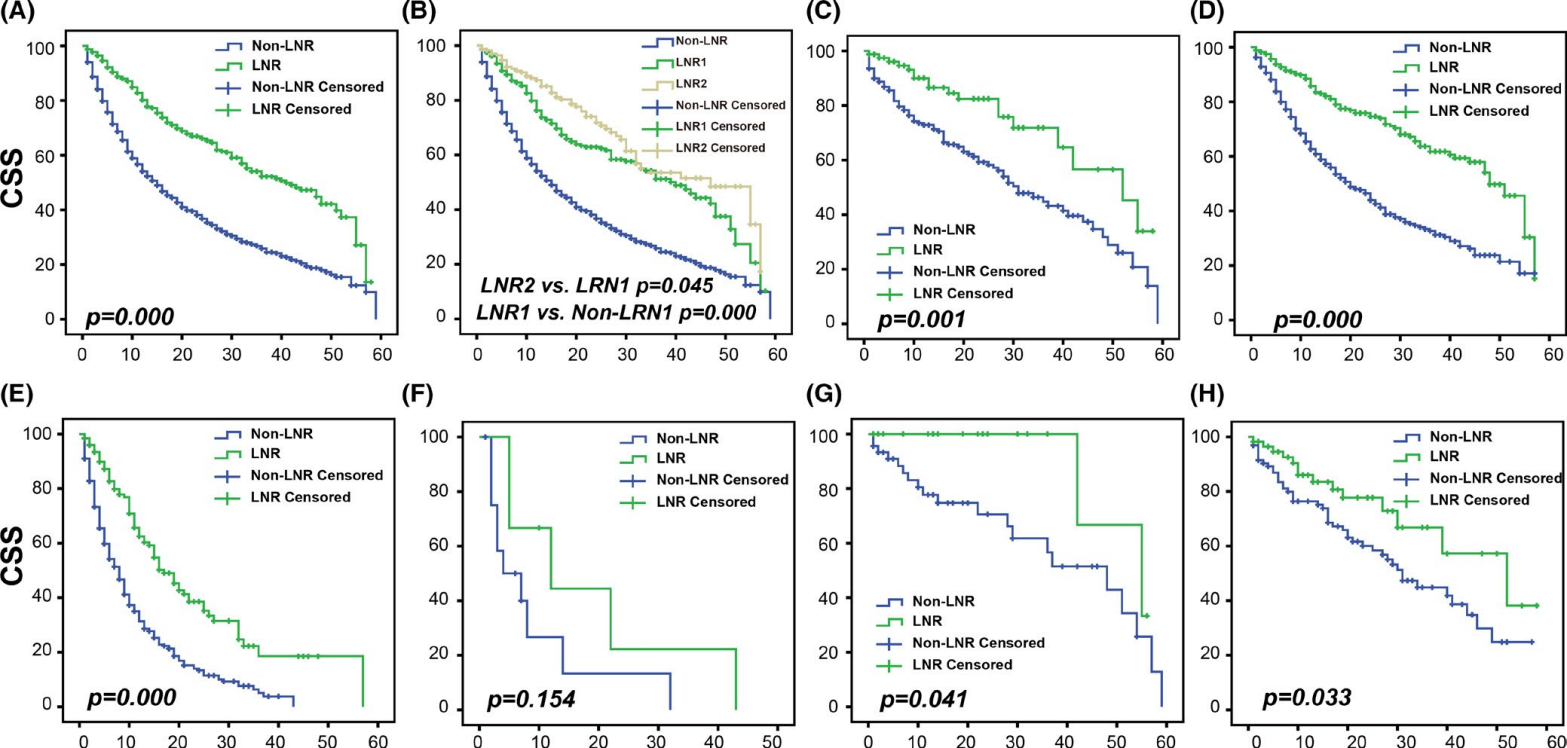
Kaplan–Meier curves comparing the LNR group with the Non‐LNR group by T stage. (A) Comparison between the LNR group and Non‐LNR group of all GBC patients, (B) comparison between the Non‐LNR group, LNR1 group, and LNR2 group of all GBC patients, (C) comparison between the LNR group and Non‐LNR group of T1 GBC patients, (D) comparison between the LNR group and Non‐LNR group of T2 GBC patients, (E) comparison between the LNR group and Non‐LNR group of T3 GBC patients, (F) comparison between the LNR group and Non‐LNR group of T4 GBC patients, (G) comparison between the LNR group and Non‐LNR group of T1a GBC patients, and (H) comparison between the LNR group and Non‐LNR group of T1b GBC patients. CSS, cancer‐specific survival; GBC, gallbladder carcinoma; LNR, lymph nodes removal

The median CSS for N0 GBC patients was 41 (32.56–49.44) months in the LNR group and 15 (12.98–17.02) months in the Non‐LNR group, with a statistically significant difference in *p* = 0.000 (Figure 2A). In addition, the comparison between the Non‐LNR group, LNR1 group, and LNR2 group (Figure 2B) show that the CSS of the LNR1 group was better than that of the Non‐LNR group (40 vs. 15 months, *p* = 0.000) significantly. Additionally, the extended lymph node dissection group (LNR2) further increased the CSS over LNR1 (47 vs. 40 months, *p* = 0.045).

### 3.3 Effect of LNR on CSS of N0 GBC patients by T stage

To determine the impact of LNR in N0 GBC patients by T stage, Kaplan–Meier analyses were performed in different T stages. In the subgroups of T1N0, T2N0, and T3N0, CSS was greater in the LNR group than in the Non‐LNR group (*p* = 0.001, 0.000, 0.000, Figure 2C–E). However, in the subgroup of T4N0, there was no significant difference between the two groups of CSS (*p* = 0.154, Figure 2F), indicating that LNR cannot extend the survival time of N0 GBC patients. Interestingly, we found that for early GBC patients in stages T1aN0 and T1bN0, LNR can extend the survival time of patients after surgery (Figure 2G,H).

### 3.4 Univariate and Cox multivariate survival analyses in N0 GBC patients

Univariate analysis showed that T stage, age, M stage, histological differentiation, liver metastasis, and LNR were significantly associated with the prognosis of N0 GBC patients (*p* < 0.05). Variables with a significant correlation with survival were included in the Cox multivariate survival analysis. The results showed that LNR is an independent prognostic factor in N0 GBC patients. The risk HR in LNR2 and LNR1 patients for the Non‐LNR group was 0.440 and 0.572, respectively (*p* = 0.000), indicating that LNR is a favorable independent prognostic factor (Table 2).

**TABLE 2:** Univariate and multivariate analyses of N0 gallbladder carcinoma patients

	Univariate analysis	Multivariate analysis	
	*p* value	HR (95% CI)	*p* value
Age, years	0.000	1.018 (1.011–1.025)	0.000
Race (Black/Other/White)	0.942		
Sex (female/male)	0.290		
T	0.000		
T1		Reference	
T2		1.235 (0.983–1.553)	0.070
T3		2.663 (2.096–3.384)	0.000
T4		3.805 (2.200–6.582)	0.000
M	0.000		
M0		Reference	
M1		2.503 (1.924–3.254)	0.000
Grade, *n* (%)	0.000		
Well differentiated I		Reference	
Moderately differentiated II		1.153 (0.925–1.435)	0.205
Poorly or undifferentiated III/IV		1.793 (1.428–2.252)	0.000
Liver metastasis	0.000		
No		Reference	
Yes		0.958 (0.705–1.302)	0.784
LNR	0.000		
Non‐LNR		Reference	
LNR1		0.572 (0.467–0.700)	0.000
LNR2		0.440 (0.332–0.584)	0.000

Abbreviation: LNR, lymph nodes removal.

### 3.5 Nomogram of N0 GBC patients

**FIGURE 3:**
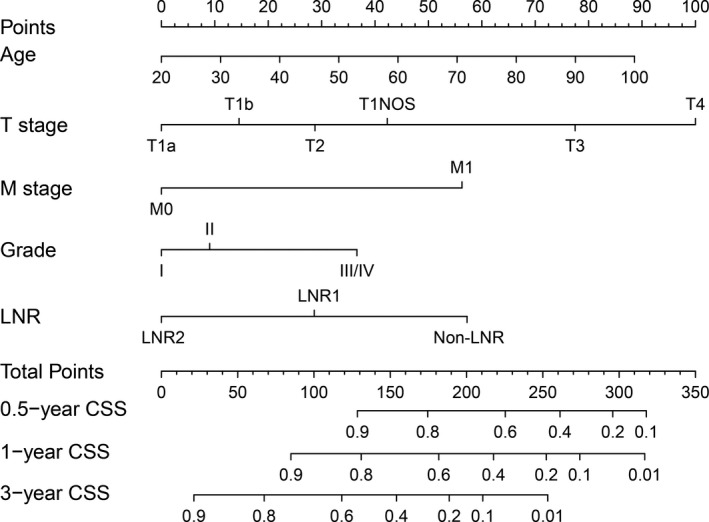
Nomogram predicting 0.5‐, 1‐, and 3‐year CSS for N0 GBC patients. The nomogram was used by accumulating the points identified on the points scale for each variable. Based on the sum of these points projected on the bottom scales, the nomogram can provide the probability of 0.5‐, 1‐, and 3‐year CSS for an individual patient. CSS, cancer‐specific survival; GBC, gallbladder carcinoma; LNR, lymph nodes removal

The nomogram for predicting 0.5‐, 1‐, and 3‐year CSS (Figure 3) based on the significant risk factors was identified by Cox multivariate analysis. To calculate the 0.5‐, 1‐, and 3‐year CSS rates, we need to determine each factor based on the points scale at the top of the nomogram first. The points for each factor were then added up. Finally, the OS rates (3 or 5 year) were obtained based on point scale on the bottom of the nomogram. According to the nomogram, the T1a score was 0, the T1b score was 15, the T2 score was 28.75, the T3 score was 77.5, and the T4 score was 100; the M0 score was 0 and the M1 score was 56.25; the histological grade Grade I score was 0, Grade II was 10, and Grade III/IV was 36.25; the LNR2 score was 0, LNR1 was 28.75, and Non‐LNR was 57.5. The LNR2 and LNR1 scores were lower than the Non‐LNR score, which shows that LNR is favorable to CSS of N0 GBC patients.

**FIGURE 4:**
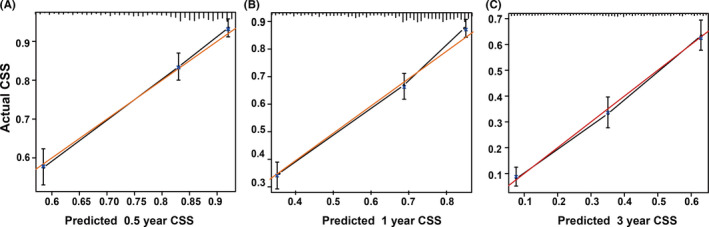
The calibration curve for predicting patients’ CSS at 0.5 year (A), 1 year (B), and 3 year (C). The *X*‐axis represented the nomogram predicted survival and the *Y*‐axis represented the actual survival. The red line represented the ideal correlation between the nomogram that was predicted and actual survival. CSS, cancer‐specific survival

The *C*‐index for prediction of 3‐year CSS was 0.735, which was higher than the *C*‐index according to TM staging in the seventh edition of the AJCC (*C*‐index = 0.678) for N0 GBC patients. It showed that the nomogram of N0 GBC patients is in good agreement with the actual prediction of the CSS rates. Figure 4 shows the calibration plot based on bootstrap resampling validation. The plots showed that the predicted CSS of nomogram corresponded to the actual survival as estimated by the Kaplan–Meier method closely.

## 4 DISCUSSION

Surgical treatment is the most important treatment of GBC, and the necessity of lymph node dissection and the extent of lymphadenectomy has been the focus of controversy. It remains unclear whether lymph node dissection is necessary for patients with N0 GBC. Most of the past studies suggest that for T1a GBC without lymph node metastasis, only simple cholecystectomy without the need for additional lymph node dissection was generally sufficient.^2^, ^20^ In this study, we demonstrated that a greater number of LNR correlated with better survival of N0 GBC patients, including those in the T1 stage.

We found that only 12.5% of N0 patients had 4 or more lymph nodes removed, whereas 64.5% had no lymph nodes removed and 22.9% had localized lymph nodes removed (1–3 nodes removed) from 2010 to 2014. In recent years, there has been a small increase in the number of lymph node dissections in N0 GBC, which reached 38.1% in 2014. The above data suggested that people are not well aware of the benefits of lymph node dissection for the prognosis of patients with N0 GBC.

The results showed that cholecystectomy with LNR1 or LNR2 can improve the survival of N0 GBC patients by Kaplan–Meier survival analysis, and a wider range of lymph node dissection (LNR2) compared to LNR1 is more conducive to the prognosis. This suggests that aggressive, more radical lymphadenectomy is beneficial for N0 GBC patients. In addition, in the subgroup analysis by T staging, LNR could also play a positive role in early patients with T1a and T1b. This contrasts the previous study that suggested that T1a and T1b without lymphatic metastasis only need simple cholecystectomy, without the need for other operations.^21^ They think that if there are one or more lymph nodes in the final specimen that were pathologically identified, regional lymphadenectomy will be taken.^22^ Our results may change our previous insights into surgical strategies for patients with early‐stage GBC. Further multivariate regression analysis showed that LNR was an independent favorable prognostic factor of N0 GBC. For patients who did not undergo lymph node dissection, the risk of death from those who undergo the operation for LNR1 or LNR2 was 0.572 or 0.440, respectively. The nomogram shows an LNR2 score of 0, an LNR1 score of 28.75, and a Non‐LNR score of 57.5. This indicates that LNR does reduce the risk of death in N0 GBC patients.

The results of this study questioned why the excision of lymph nodes that are not infiltrated by tumor cells is beneficial to the patient's prognosis and whether these lymph nodes are really unaffected by the tumor. There are three main reasons to explain this phenomenon. First, the lymphatic drainage of the gallbladder is very complex and varied,^23^ and routine sentinel lymph node examinations may not involve all possible pathways, resulting in the omission of positive lymph nodes. Second, tumor cells are not the only form of tumor metastasis. Tumors may metastasize in the form of free DNA (ctDNA) or RNA.^24^ Routine pathology does not effectively differentiate lymph nodes that are affected by the tumor, which may yield false positives if they are negative lymph nodes. Third and most importantly, this study suggests that the tumor microenvironment plays a crucial role in the development of the tumor. Tumor microenvironment includes infiltrating lymphocytes, stromal cells, chemokines, hypoxia microenvironment, and so on. Research suggests that the local tissue microenvironment may have changed before the tumor appeared, a condition that favors tumorigenesis.^25^, ^26^, ^27^ The phenomenon most likely also occurs in the lymph nodes. Free tumor genetic material (ctDNA and RNA) in such an environment caused tumor recurrence and metastasis. Therefore, radical resection of lymph nodes is advantageous, although lymph nodes have not yet been invaded by tumors cells in N0 GBC patients. Of course, these are just our speculations about this phenomenon, and more research is essential.

There are some limitations to this study. Whether patients received other treatment (i.e., chemotherapy, biliary tree reconstruction, etc.) and information of recurrence are not available for GBC in the database of SEER. Even though the location of the tumor on the gallbladder (the side of the gallbladder near the liver or away from the liver) is not available in the current database. Nevertheless, our analysis provides generalizable and credible results based on more than 1400 N0 GBC patients. This shows that our conclusion is a more accurate basis for the surgical approach.

## 5 CONCLUSIONS

In conclusion, this study demonstrated a significant survival benefit for extended lymph nodes removed in N0 GBC patients. These results recommend that an extended lymph node dissection strategy is needed for N0 GBC patients. Furthermore, we established a prognostic model of survival for N0 GBC patients based on the large samples.

## CONFLICT OF INTEREST

The authors have no conflict of interest to declare.

## RESEARCH INVOLVING HUMAN PARTICIPANTS AND/OR ANIMALS

This article does not contain any studies with animals or human participants performed by any of the authors.

## Data Availability

The data that support the findings of this study are available upon request from the corresponding author. The data are not publicly available due to privacy or ethical restrictions.
